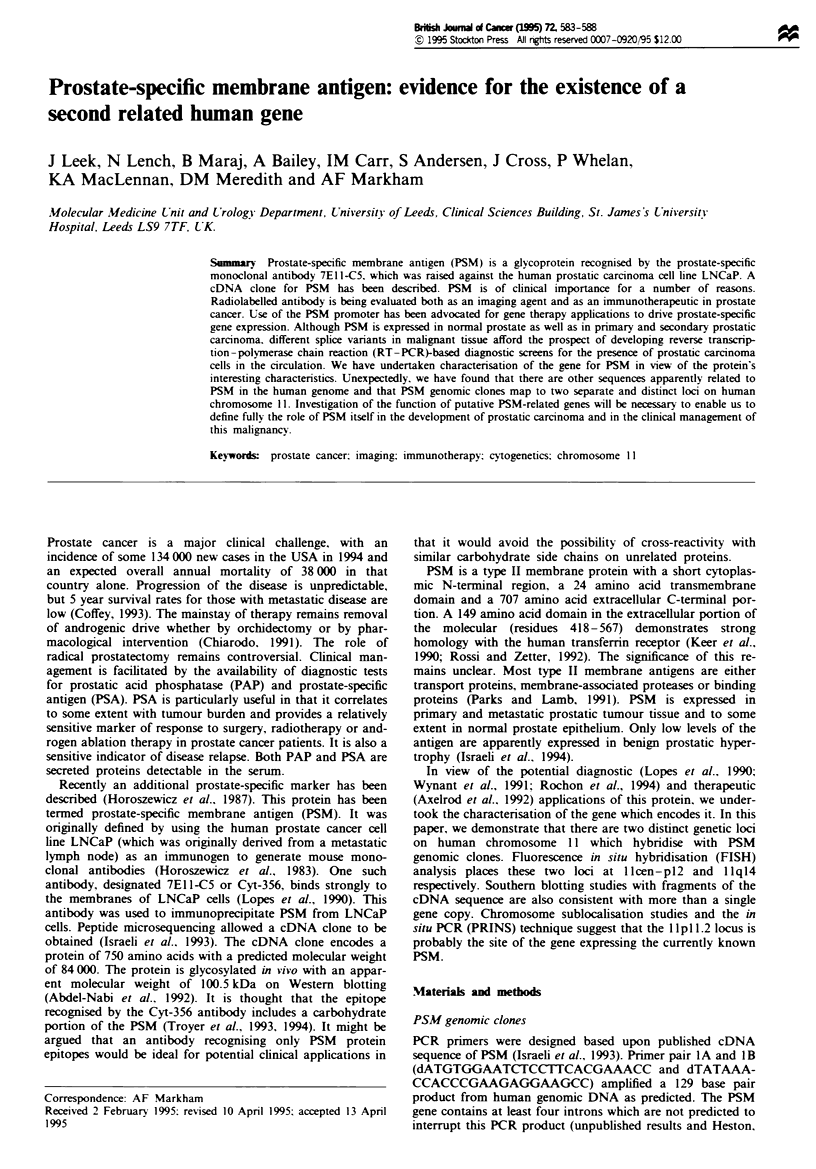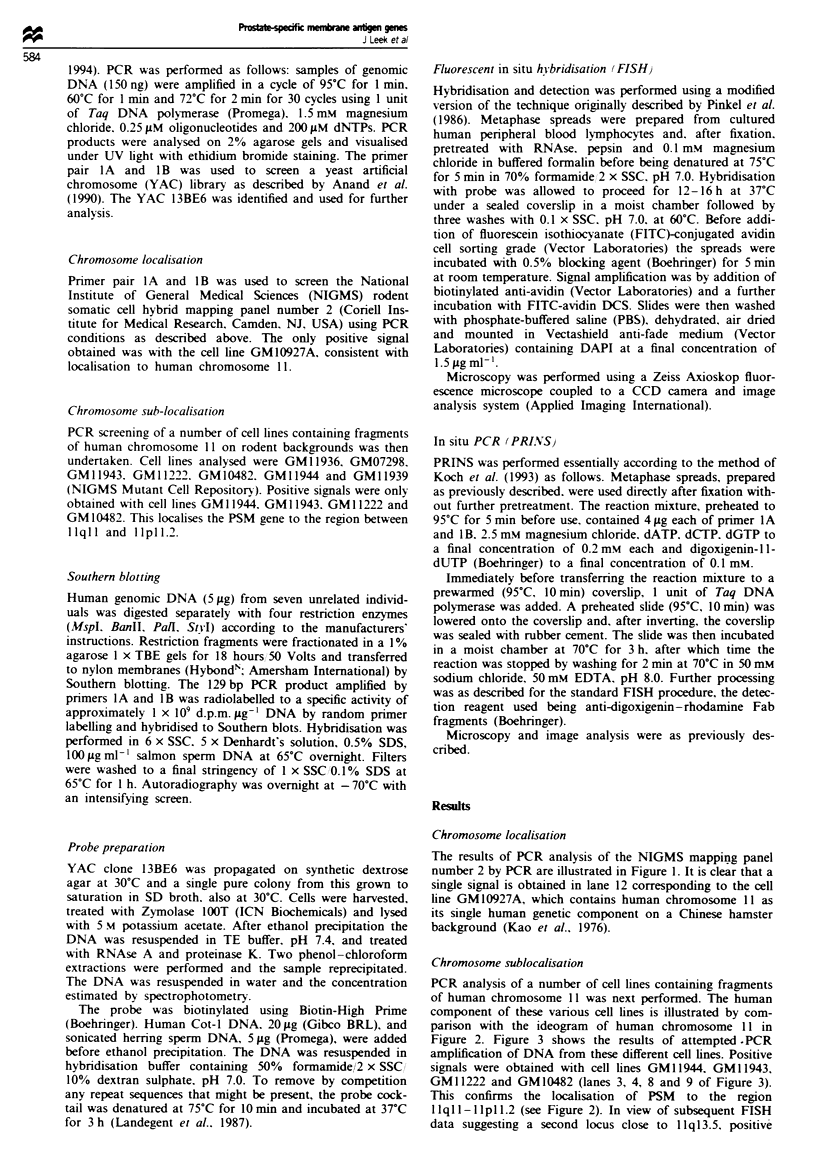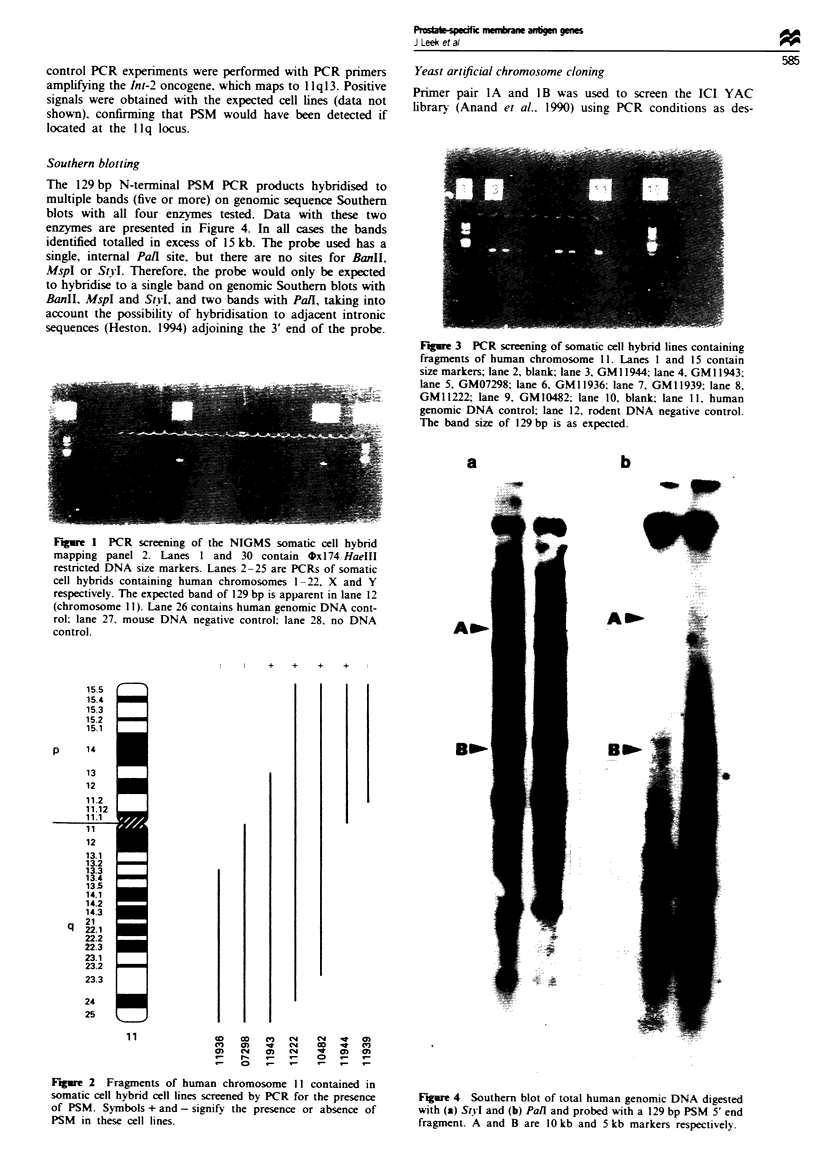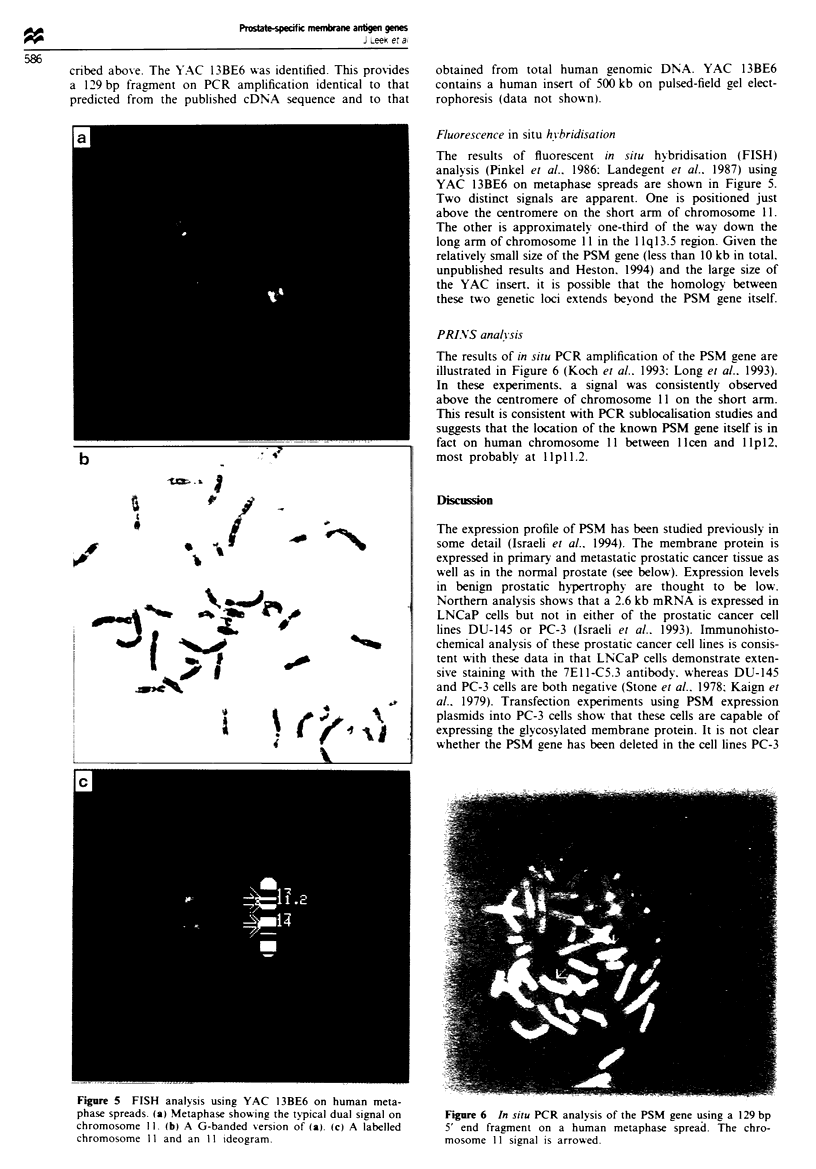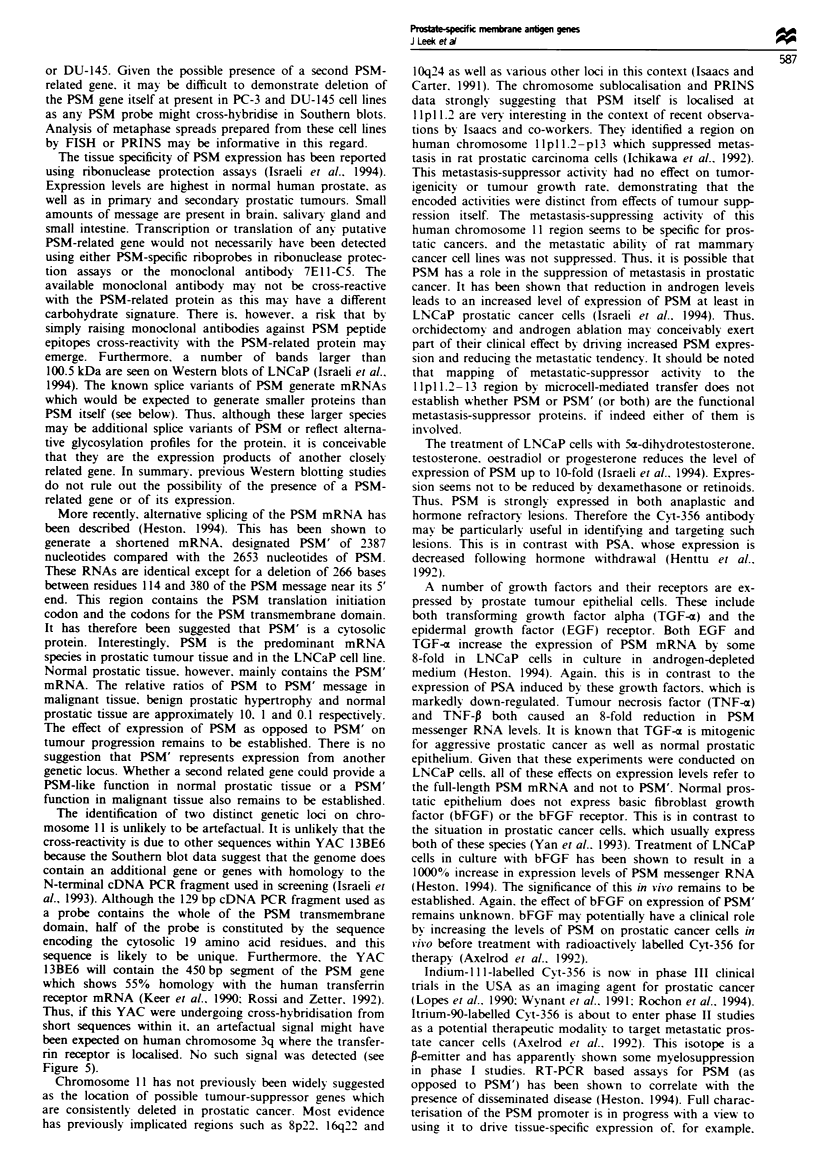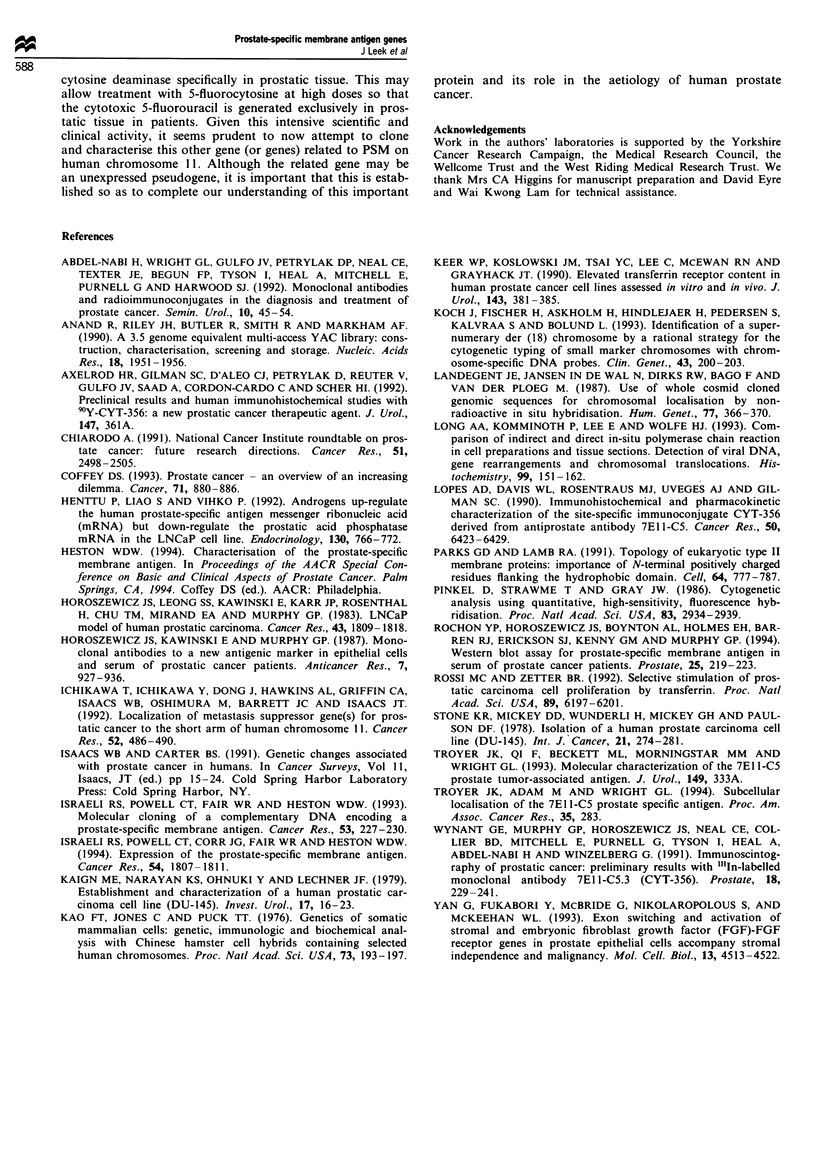# Prostate-specific membrane antigen: evidence for the existence of a second related human gene.

**DOI:** 10.1038/bjc.1995.377

**Published:** 1995-09

**Authors:** J. Leek, N. Lench, B. Maraj, A. Bailey, I. M. Carr, S. Andersen, J. Cross, P. Whelan, K. A. MacLennan, D. M. Meredith

**Affiliations:** Molecular Medicine Unit, University of Leeds, UK.

## Abstract

**Images:**


British Journal of Cancer (1995) 72, 583-588

© 1995 Stockton Press All rights reserved 0007-0920/95 $12.00

### Prostate-specific membrane antigen: evidence for the existence of a second related human gene

J Leek, N Lench, B Maraj, A Bailey, IM Carr, S Andersen, J Cross, P Whelan, KA MacLennan, DM Meredith and AF Markham

*Molecular Medicine Unit and Urology Department, University of Leeds, Clinical Sciences Building, St. James's University Hospital, Leeds LS9 7TF, UK.*

**Summary** Prostate-specific membrane antigen (PSM) is a glycoprotein recognised by the prostate-specific monoclonal antibody 7E11-C5, which was raised against the human prostatic carcinoma cell line LNCaP. A cDNA clone for PSM has been described. PSM is of clinical importance for a number of reasons. Radiolabelled antibody is being evaluated both as an imaging agent and as an immunotherapeutic in prostate cancer. Use of the PSM promoter has been advocated for gene therapy applications to drive prostate-specific gene expression. Although PSM is expressed in normal prostate as well as in primary and secondary prostatic carcinoma, different splice variants in malignant tissue afford the prospect of developing reverse transcription-polymerase chain reaction (RT-PCR)-based diagnostic screens for the presence of prostatic carcinoma cells in the circulation. We have undertaken characterisation of the gene for PSM in view of the protein's interesting characteristics. Unexpectedly, we have found that there are other sequences apparently related to PSM in the human genome and that PSM genomic clones map to two separate and distinct loci on human chromosome 11. Investigation of the function of putative PSM-related genes will be necessary to enable us to define fully the role of PSM itself in the development of prostatic carcinoma and in the clinical management of this malignancy.

**Keywords:** prostate cancer; imaging; immunotherapy; cytogenetics; chromosome 11

Prostate cancer is a major clinical challenge, with an incidence of some 134 000 new cases in the USA in 1994 and an expected overall annual mortality of 38 000 in that country alone. Progression of the disease is unpredictable, but 5 year survival rates for those with metastatic disease are low (Coffey, 1993). The mainstay of therapy remains removal of androgenic drive whether by orchidectomy or by pharmacological intervention (Chiarodo, 1991). The role of radical prostatectomy remains controversial. Clinical management is facilitated by the availability of diagnostic tests for prostatic acid phosphatase (PAP) and prostate-specific antigen (PSA). PSA is particularly useful in that it correlates to some extent with tumour burden and provides a relatively sensitive marker of response to surgery, radiotherapy or androgen ablation therapy in prostate cancer patients. It is also a sensitive indicator of disease relapse. Both PAP and PSA are secreted proteins detectable in the serum.

Recently an additional prostate-specific marker has been described (Horoszewicz *et al.*, 1987). This protein has been termed prostate-specific membrane antigen (PSM). It was originally defined by using the human prostate cancer cell line LNCaP (which was originally derived from a metastatic lymph node) as an immunogen to generate mouse monoclonal antibodies (Horoszewicz *et al.*, 1983). One such antibody, designated 7E11-C5 or Cyt-356, binds strongly to the membranes of LNCaP cells (Lopes *et al.*, 1990). This antibody was used to immunoprecipitate PSM from LNCaP cells. Peptide microsequencing allowed a cDNA clone to be obtained (Israeli *et al.*, 1993). The cDNA clone encodes a protein of 750 amino acids with a predicted molecular weight of 84 000. The protein is glycosylated *in vivo* with an apparent molecular weight of 100.5 kDa on Western blotting (Abdel-Nabi *et al.*, 1992). It is thought that the epitope recognised by the Cyt-356 antibody includes a carbohydrate portion of the PSM (Troyer *et al.*, 1993, 1994). It might be argued that an antibody recognising only PSM protein epitopes would be ideal for potential clinical applications in that it would avoid the possibility of cross-reactivity with similar carbohydrate side chains on unrelated proteins.

PSM is a type II membrane protein with a short cytoplasmic N-terminal region, a 24 amino acid transmembrane domain and a 707 amino acid extracellular C-terminal portion. A 149 amino acid domain in the extracellular portion of the molecular (residues 418-567) demonstrates strong homology with the human transferrin receptor (Keer *et al.*, 1990; Rossi and Zetter, 1992). The significance of this remains unclear. Most type II membrane antigens are either transport proteins, membrane-associated proteases or binding proteins (Parks and Lamb, 1991). PSM is expressed in primary and metastatic prostatic tumour tissue and to some extent in normal prostate epithelium. Only low levels of the antigen are apparently expressed in benign prostatic hypertrophy (Israeli *et al.*, 1994).

In view of the potential diagnostic (Lopes *et al.*, 1990; Wynant *et al.*, 1991; Rochon *et al.*, 1994) and therapeutic (Axelrod *et al.*, 1992) applications of this protein, we undertook the characterisation of the gene which encodes it. In this paper, we demonstrate that there are two distinct genetic loci on human chromosome 11 which hybridise with PSM genomic clones. Fluorescence *in situ* hybridisation (FISH) analysis places these two loci at 11cen-p12 and 11q14 respectively. Southern blotting studies with fragments of the cDNA sequence are also consistent with more than a single gene copy. Chromosome sublocalisation studies and the *in situ* PCR (PRINS) technique suggest that the 11p11.2 locus is probably the site of the gene expressing the currently known PSM.

### Materials and methods

### *PSM genomic clones*

PCR primers were designed based upon published cDNA sequence of PSM (Israeli *et al.*, 1993). Primer pair 1A and 1B (dATGTGGAATCTCCTTCACGAAACC and dTATAAACCACCCGAAGAGGAAGCC) amplified a 129 base pair product from human genomic DNA as predicted. The PSM gene contains at least four introns which are not predicted to interrupt this PCR product (unpublished results and Heston,

Correspondence: AF Markham
Received 2 February 1995; revised 10 April 1995; accepted 13 April 1995

1994). PCR was performed as follows: samples of genomic DNA (150 ng) were amplified in a cycle of 95°C for 1 min, 60°C for 1 min and 72°C for 2 min for 30 cycles using 1 unit of *Taq* DNA polymerase (Promega), 1.5 mM magnesium chloride, 0.25 μM oligonucleotides and 200 μM dNTPs. PCR products were analysed on 2% agarose gels and visualised under UV light with ethidium bromide staining. The primer pair 1A and 1B was used to screen a yeast artificial chromosome (YAC) library as described by Anand *et al.* (1990). The YAC 13BE6 was identified and used for further analysis.

*Chromosome localisation*

Primer pair 1A and 1B was used to screen the National Institute of General Medical Sciences (NIGMS) rodent somatic cell hybrid mapping panel number 2 (Coriell Institute for Medical Research, Camden, NJ, USA) using PCR conditions as described above. The only positive signal obtained was with the cell line GM10927A, consistent with localisation to human chromosome 11.

*Chromosome sub-localisation*

PCR screening of a number of cell lines containing fragments of human chromosome 11 on rodent backgrounds was then undertaken. Cell lines analysed were GM11936, GM07298, GM11943, GM11222, GM10482, GM11944 and GM11939 (NIGMS Mutant Cell Repository). Positive signals were only obtained with cell lines GM11944, GM11943, GM11222 and GM10482. This localises the PSM gene to the region between 11q11 and 11p11.2.

*Southern blotting*

Human genomic DNA (5 μg) from seven unrelated individuals was digested separately with four restriction enzymes (*Msp*I, *Ban*II, *Pal*I, *Sty*I) according to the manufacturers' instructions. Restriction fragments were fractionated in a 1% agarose 1 × TBE gels for 18 hours/50 Volts and transferred to nylon membranes (Hybond$^{N}$; Amersham International) by Southern blotting. The 129 bp PCR product amplified by primers 1A and 1B was radiolabelled to a specific activity of approximately $1 \times 10^9$ d.p.m. $\mu g^{-1}$ DNA by random primer labelling and hybridised to Southern blots. Hybridisation was performed in 6 × SSC, 5 × Denhardt's solution, 0.5% SDS, 100 $\mu g\ ml^{-1}$ salmon sperm DNA at 65°C overnight. Filters were washed to a final stringency of 1 × SSC/0.1% SDS at 65°C for 1 h. Autoradiography was overnight at −70°C with an intensifying screen.

*Probe preparation*

YAC clone 13BE6 was propagated on synthetic dextrose agar at 30°C and a single pure colony from this grown to saturation in SD broth, also at 30°C. Cells were harvested, treated with Zymolase 100T (ICN Biochemicals) and lysed with 5 M potassium acetate. After ethanol precipitation the DNA was resuspended in TE buffer, pH 7.4, and treated with RNAse A and proteinase K. Two phenol–chloroform extractions were performed and the sample reprecipitated. The DNA was resuspended in water and the concentration estimated by spectrophotometry.

The probe was biotinylated using Biotin-High Prime (Boehringer). Human Cot-1 DNA, 20 μg (Gibco BRL), and sonicated herring sperm DNA, 5 μg (Promega), were added before ethanol precipitation. The DNA was resuspended in hybridisation buffer containing 50% formamide/2 × SSC/10% dextran sulphate, pH 7.0. To remove by competition any repeat sequences that might be present, the probe cocktail was denatured at 75°C for 10 min and incubated at 37°C for 3 h (Landegent *et al.*, 1987).

*Fluorescent* in situ *hybridisation (FISH)*

Hybridisation and detection was performed using a modified version of the technique originally described by Pinkel *et al.* (1986). Metaphase spreads were prepared from cultured human peripheral blood lymphocytes and, after fixation, pretreated with RNAse, pepsin and 0.1 mM magnesium chloride in buffered formalin before being denatured at 75°C for 5 min in 70% formamide/2 × SSC, pH 7.0. Hybridisation with probe was allowed to proceed for 12–16 h at 37°C under a sealed coverslip in a moist chamber followed by three washes with 0.1 × SSC, pH 7.0, at 60°C. Before addition of fluorescein isothiocyanate (FITC)-conjugated avidin cell sorting grade (Vector Laboratories) the spreads were incubated with 0.5% blocking agent (Boehringer) for 5 min at room temperature. Signal amplification was by addition of biotinylated anti-avidin (Vector Laboratories) and a further incubation with FITC-avidin DCS. Slides were then washed with phosphate-buffered saline (PBS), dehydrated, air dried and mounted in Vectashield anti-fade medium (Vector Laboratories) containing DAPI at a final concentration of 1.5 $\mu g\ ml^{-1}$.

Microscopy was performed using a Zeiss Axioskop fluorescence microscope coupled to a CCD camera and image analysis system (Applied Imaging International).

In situ *PCR (PRINS)*

PRINS was performed essentially according to the method of Koch *et al.* (1993) as follows. Metaphase spreads, prepared as previously described, were used directly after fixation without further pretreatment. The reaction mixture, preheated to 95°C for 5 min before use, contained 4 μg each of primer 1A and 1B, 2.5 mM magnesium chloride, dATP, dCTP, dGTP to a final concentration of 0.2 mM each and digoxigenin-11-dUTP (Boehringer) to a final concentration of 0.1 mM.

Immediately before transferring the reaction mixture to a prewarmed (95°C, 10 min) coverslip, 1 unit of *Taq* DNA polymerase was added. A preheated slide (95°C, 10 min) was lowered onto the coverslip and, after inverting, the coverslip was sealed with rubber cement. The slide was then incubated in a moist chamber at 70°C for 3 h, after which time the reaction was stopped by washing for 2 min at 70°C in 50 mM sodium chloride, 50 mM EDTA, pH 8.0. Further processing was as described for the standard FISH procedure, the detection reagent used being anti-digoxigenin–rhodamine Fab fragments (Boehringer).

Microscopy and image analysis were as previously described.

### Results

*Chromosome localisation*

The results of PCR analysis of the NIGMS mapping panel number 2 by PCR are illustrated in Figure 1. It is clear that a single signal is obtained in lane 12 corresponding to the cell line GM10927A, which contains human chromosome 11 as its single human genetic component on a Chinese hamster background (Kao *et al.*, 1976).

*Chromosome sublocalisation*

PCR analysis of a number of cell lines containing fragments of human chromosome 11 was next performed. The human component of these various cell lines is illustrated by comparison with the ideogram of human chromosome 11 in Figure 2. Figure 3 shows the results of attempted PCR amplification of DNA from these different cell lines. Positive signals were obtained with cell lines GM11944, GM11943, GM11222 and GM10482 (lanes 3, 4, 8 and 9 of Figure 3). This confirms the localisation of PSM to the region 11q11–11p11.2 (see Figure 2). In view of subsequent FISH data suggesting a second locus close to 11q13.5, positive control PCR experiments were performed with PCR primers amplifying the *Int*-2 oncogene, which maps to 11q13. Positive signals were obtained with the expected cell lines (data not shown), confirming that PSM would have been detected if located at the 11q locus.

*Southern blotting*

The 129 bp N-terminal PSM PCR products hybridised to multiple bands (five or more) on genomic sequence Southern blots with all four enzymes tested. Data with these two enzymes are presented in Figure 4. In all cases the bands identified totalled in excess of 15 kb. The probe used has a single, internal *PalI* site, but there are no sites for *BanII*, *MspI* or *StyI*. Therefore, the probe would only be expected to hybridise to a single band on genomic Southern blots with *BanII*, *MspI* and *StyI*, and two bands with *PalI*, taking into account the possibility of hybridisation to adjacent intronic sequences (Heston, 1994) adjoining the 3′ end of the probe.

**Figure 1** PCR screening of the NIGMS somatic cell hybrid mapping panel 2. Lanes 1 and 30 contain Φx174/*HaeIII* restricted DNA size markers. Lanes 2–25 are PCRs of somatic cell hybrids containing human chromosomes 1–22, X and Y respectively. The expected band of 129 bp is apparent in lane 12 (chromosome 11). Lane 26 contains human genomic DNA control; lane 27, mouse DNA negative control; lane 28, no DNA control.

**Figure 2** Fragments of human chromosome 11 contained in somatic cell hybrid cell lines screened by PCR for the presence of PSM. Symbols + and – signify the presence or absence of PSM in these cell lines.

*Yeast artificial chromosome cloning*

Primer pair 1A and 1B was used to screen the ICI YAC library (Anand *et al*., 1990) using PCR conditions as des-

**Figure 3** PCR screening of somatic cell hybrid lines containing fragments of human chromosome 11. Lanes 1 and 15 contain size markers; lane 2, blank; lane 3, GM11944; lane 4, GM11943; lane 5, GM07298; lane 6, GM11936; lane 7, GM11939; lane 8, GM11222; lane 9, GM10482; lane 10, blank; lane 11, human genomic DNA control; lane 12, rodent DNA negative control. The band size of 129 bp is as expected.

**Figure 4** Southern blot of total human genomic DNA digested with (**a**) *StyI* and (**b**) *PalI* and probed with a 129 bp PSM 5′ end fragment. A and B are 10 kb and 5 kb markers respectively.

cribed above. The YAC 13BE6 was identified. This provides a 129 bp fragment on PCR amplification identical to that predicted from the published cDNA sequence and to that obtained from total human genomic DNA. YAC 13BE6 contains a human insert of 500 kb on pulsed-field gel electrophoresis (data not shown).

Figure 5 FISH analysis using YAC 13BE6 on human metaphase spreads. (a) Metaphase showing the typical dual signal on chromosome 11. (b) A G-banded version of (a). (c) A labelled chromosome 11 and an 11 ideogram.

### *Fluorescence* in situ *hybridisation*

The results of fluorescent *in situ* hybridisation (FISH) analysis (Pinkel *et al*., 1986; Landegent *et al*., 1987) using YAC 13BE6 on metaphase spreads are shown in Figure 5. Two distinct signals are apparent. One is positioned just above the centromere on the short arm of chromosome 11. The other is approximately one-third of the way down the long arm of chromosome 11 in the 11q13.5 region. Given the relatively small size of the PSM gene (less than 10 kb in total, unpublished results and Heston, 1994) and the large size of the YAC insert, it is possible that the homology between these two genetic loci extends beyond the PSM gene itself.

### *PRINS analysis*

The results of *in situ* PCR amplification of the PSM gene are illustrated in Figure 6 (Koch *et al*., 1993; Long *et al*., 1993). In these experiments, a signal was consistently observed above the centromere of chromosome 11 on the short arm. This result is consistent with PCR sublocalisation studies and suggests that the location of the known PSM gene itself is in fact on human chromosome 11 between 11cen and 11p12, most probably at 11p11.2.

### Discussion

The expression profile of PSM has been studied previously in some detail (Israeli *et al*., 1994). The membrane protein is expressed in primary and metastatic prostatic cancer tissue as well as in the normal prostate (see below). Expression levels in benign prostatic hypertrophy are thought to be low. Northern analysis shows that a 2.6 kb mRNA is expressed in LNCaP cells but not in either of the prostatic cancer cell lines DU-145 or PC-3 (Israeli *et al*., 1993). Immunohistochemical analysis of these prostatic cancer cell lines is consistent with these data in that LNCaP cells demonstrate extensive staining with the 7E11-C5.3 antibody, whereas DU-145 and PC-3 cells are both negative (Stone *et al*., 1978; Kaign *et al*., 1979). Transfection experiments using PSM expression plasmids into PC-3 cells show that these cells are capable of expressing the glycosylated membrane protein. It is not clear whether the PSM gene has been deleted in the cell lines PC-3

Figure 6 *In situ* PCR analysis of the PSM gene using a 129 bp 5′ end fragment on a human metaphase spread. The chromosome 11 signal is arrowed.

or DU-145. Given the possible presence of a second PSM-related gene, it may be difficult to demonstrate deletion of the PSM gene itself at present in PC-3 and DU-145 cell lines as any PSM probe might cross-hybridise in Southern blots. Analysis of metaphase spreads prepared from these cell lines by FISH or PRINS may be informative in this regard.

The tissue specificity of PSM expression has been reported using ribonuclease protection assays (Israeli *et al.*, 1994). Expression levels are highest in normal human prostate, as well as in primary and secondary prostatic tumours. Small amounts of message are present in brain, salivary gland and small intestine. Transcription or translation of any putative PSM-related gene would not necessarily have been detected using either PSM-specific riboprobes in ribonuclease protection assays or the monoclonal antibody 7E11-C5. The available monoclonal antibody may not be cross-reactive with the PSM-related protein as this may have a different carbohydrate signature. There is, however, a risk that by simply raising monoclonal antibodies against PSM peptide epitopes cross-reactivity with the PSM-related protein may emerge. Furthermore, a number of bands larger than 100.5 kDa are seen on Western blots of LNCaP (Israeli *et al.*, 1994). The known splice variants of PSM generate mRNAs which would be expected to generate smaller proteins than PSM itself (see below). Thus, although these larger species may be additional splice variants of PSM or reflect alternative glycosylation profiles for the protein, it is conceivable that they are the expression products of another closely related gene. In summary, previous Western blotting studies do not rule out the possibility of the presence of a PSM-related gene or of its expression.

More recently, alternative splicing of the PSM mRNA has been described (Heston, 1994). This has been shown to generate a shortened mRNA, designated PSM′ of 2387 nucleotides compared with the 2653 nucleotides of PSM. These RNAs are identical except for a deletion of 266 bases between residues 114 and 380 of the PSM message near its 5′ end. This region contains the PSM translation initiation codon and the codons for the PSM transmembrane domain. It has therefore been suggested that PSM′ is a cytosolic protein. Interestingly, PSM is the predominant mRNA species in prostatic tumour tissue and in the LNCaP cell line. Normal prostatic tissue, however, mainly contains the PSM′ mRNA. The relative ratios of PSM to PSM′ message in malignant tissue, benign prostatic hypertrophy and normal prostatic tissue are approximately 10, 1 and 0.1 respectively. The effect of expression of PSM as opposed to PSM′ on tumour progression remains to be established. There is no suggestion that PSM′ represents expression from another genetic locus. Whether a second related gene could provide a PSM-like function in normal prostatic tissue or a PSM′ function in malignant tissue also remains to be established.

The identification of two distinct genetic loci on chromosome 11 is unlikely to be artefactual. It is unlikely that the cross-reactivity is due to other sequences within YAC 13BE6 because the Southern blot data suggest that the genome does contain an additional gene or genes with homology to the N-terminal cDNA PCR fragment used in screening (Israeli *et al.*, 1993). Although the 129 bp cDNA PCR fragment used as a probe contains the whole of the PSM transmembrane domain, half of the probe is constituted by the sequence encoding the cytosolic 19 amino acid residues, and this sequence is likely to be unique. Furthermore, the YAC 13BE6 will contain the 450 bp segment of the PSM gene which shows 55% homology with the human transferrin receptor mRNA (Keer *et al.*, 1990; Rossi and Zetter, 1992). Thus, if this YAC were undergoing cross-hybridisation from short sequences within it, an artefactual signal might have been expected on human chromosome 3q where the transferrin receptor is localised. No such signal was detected (see Figure 5).

Chromosome 11 has not previously been widely suggested as the location of possible tumour-suppressor genes which are consistently deleted in prostatic cancer. Most evidence has previously implicated regions such as 8p22, 16q22 and 10q24 as well as various other loci in this context (Isaacs and Carter, 1991). The chromosome sublocalisation and PRINS data strongly suggesting that PSM itself is localised at 11p11.2 are very interesting in the context of recent observations by Isaacs and co-workers. They identified a region on human chromosome 11p11.2–p13 which suppressed metastasis in rat prostatic carcinoma cells (Ichikawa *et al.*, 1992). This metastasis-suppressor activity had no effect on tumorigenicity or tumour growth rate, demonstrating that the encoded activities were distinct from effects of tumour suppression itself. The metastasis-suppressing activity of this human chromosome 11 region seems to be specific for prostatic cancers, and the metastatic ability of rat mammary cancer cell lines was not suppressed. Thus, it is possible that PSM has a role in the suppression of metastasis in prostatic cancer. It has been shown that reduction in androgen levels leads to an increased level of expression of PSM at least in LNCaP prostatic cancer cells (Israeli *et al.*, 1994). Thus, orchidectomy and androgen ablation may conceivably exert part of their clinical effect by driving increased PSM expression and reducing the metastatic tendency. It should be noted that mapping of metastatic-suppressor activity to the 11p11.2–13 region by microcell-mediated transfer does not establish whether PSM or PSM′ (or both) are the functional metastasis-suppressor proteins, if indeed either of them is involved.

The treatment of LNCaP cells with 5α-dihydrotestosterone, testosterone, oestradiol or progesterone reduces the level of expression of PSM up to 10-fold (Israeli *et al.*, 1994). Expression seems not to be reduced by dexamethasone or retinoids. Thus, PSM is strongly expressed in both anaplastic and hormone refractory lesions. Therefore the Cyt-356 antibody may be particularly useful in identifying and targeting such lesions. This is in contrast with PSA, whose expression is decreased following hormone withdrawal (Henttu *et al.*, 1992).

A number of growth factors and their receptors are expressed by prostate tumour epithelial cells. These include both transforming growth factor alpha (TGF-α) and the epidermal growth factor (EGF) receptor. Both EGF and TGF-α increase the expression of PSM mRNA by some 8-fold in LNCaP cells in culture in androgen-depleted medium (Heston, 1994). Again, this is in contrast to the expression of PSA induced by these growth factors, which is markedly down-regulated. Tumour necrosis factor (TNF-α) and TNF-β both caused an 8-fold reduction in PSM messenger RNA levels. It is known that TGF-α is mitogenic for aggressive prostatic cancer as well as normal prostatic epithelium. Given that these experiments were conducted on LNCaP cells, all of these effects on expression levels refer to the full-length PSM mRNA and not to PSM′. Normal prostatic epithelium does not express basic fibroblast growth factor (bFGF) or the bFGF receptor. This is in contrast to the situation in prostatic cancer cells, which usually express both of these species (Yan *et al.*, 1993). Treatment of LNCaP cells in culture with bFGF has been shown to result in a 1000% increase in expression levels of PSM messenger RNA (Heston, 1994). The significance of this *in vivo* remains to be established. Again, the effect of bFGF on expression of PSM′ remains unknown. bFGF may potentially have a clinical role by increasing the levels of PSM on prostatic cancer cells *in vivo* before treatment with radioactively labelled Cyt-356 for therapy (Axelrod *et al.*, 1992).

Indium-111-labelled Cyt-356 is now in phase III clinical trials in the USA as an imaging agent for prostatic cancer (Lopes *et al.*, 1990; Wynant *et al.*, 1991; Rochon *et al.*, 1994). Itrium-90-labelled Cyt-356 is about to enter phase II studies as a potential therapeutic modality to target metastatic prostate cancer cells (Axelrod *et al.*, 1992). This isotope is a β-emitter and has apparently shown some myelosuppression in phase I studies. RT-PCR based assays for PSM (as opposed to PSM′) has been shown to correlate with the presence of disseminated disease (Heston, 1994). Full characterisation of the PSM promoter is in progress with a view to using it to drive tissue-specific expression of, for example, cytosine deaminase specifically in prostatic tissue. This may allow treatment with 5-fluorocytosine at high doses so that the cytotoxic 5-fluorouracil is generated exclusively in prostatic tissue in patients. Given this intensive scientific and clinical activity, it seems prudent to now attempt to clone and characterise this other gene (or genes) related to PSM on human chromosome 11. Although the related gene may be an unexpressed pseudogene, it is important that this is established so as to complete our understanding of this important protein and its role in the aetiology of human prostate cancer.

**Acknowledgements**
Work in the authors' laboratories is supported by the Yorkshire Cancer Research Campaign, the Medical Research Council, the Wellcome Trust and the West Riding Medical Research Trust. We thank Mrs CA Higgins for manuscript preparation and David Eyre and Wai Kwong Lam for technical assistance.

### References

ABDEL-NABI H, WRIGHT GL, GULFO JV, PETRYLAK DP, NEAL CE, TEXTER JE, BEGUN FP, TYSON I, HEAL A, MITCHELL E, PURNELL G AND HARWOOD SJ. (1992). Monoclonal antibodies and radioimmunoconjugates in the diagnosis and treatment of prostate cancer. *Semin. Urol.*, **10**, 45-54.

ANAND R, RILEY JH, BUTLER R, SMITH R AND MARKHAM AF. (1990). A 3.5 genome equivalent multi-access YAC library: construction, characterisation, screening and storage. *Nucleic. Acids Res.*, **18**, 1951-1956.

AXELROD HR, GILMAN SC, D'ALEO CJ, PETRYLAK D, REUTER V, GULFO JV, SAAD A, CORDON-CARDO C AND SCHER HI. (1992). Preclinical results and human immunohistochemical studies with $^{90}$Y-CYT-356: a new prostatic cancer therapeutic agent. *J. Urol.*, **147**, 361A.

CHIARODO A. (1991). National Cancer Institute roundtable on prostate cancer: future research directions. *Cancer Res.*, **51**, 2498-2505.

COFFEY DS. (1993). Prostate cancer – an overview of an increasing dilemma. *Cancer*, **71**, 880-886.

HENTTU P, LIAO S AND VIHKO P. (1992). Androgens up-regulate the human prostate-specific antigen messenger ribonucleic acid (mRNA) but down-regulate the prostatic acid phosphatase mRNA in the LNCaP cell line. *Endocrinology*, **130**, 766-772.

HESTON WDW. (1994). Characterisation of the prostate-specific membrane antigen. In *Proceedings of the AACR Special Conference on Basic and Clinical Aspects of Prostate Cancer. Palm Springs, CA, 1994.* Coffey DS (ed.). AACR: Philadelphia.

HOROSZEWICZ JS, LEONG SS, KAWINSKI E, KARR JP, ROSENTHAL H, CHU TM, MIRAND EA AND MURPHY GP. (1983). LNCaP model of human prostatic carcinoma. *Cancer Res.*, **43**, 1809-1818.

HOROSZEWICZ JS, KAWINSKI E AND MURPHY GP. (1987). Monoclonal antibodies to a new antigenic marker in epithelial cells and serum of prostatic cancer patients. *Anticancer Res.*, **7**, 927-936.

ICHIKAWA T, ICHIKAWA Y, DONG J, HAWKINS AL, GRIFFIN CA, ISAACS WB, OSHIMURA M, BARRETT JC AND ISAACS JT. (1992). Localization of metastasis suppressor gene(s) for prostatic cancer to the short arm of human chromosome 11. *Cancer Res.*, **52**, 486-490.

ISAACS WB AND CARTER BS. (1991). Genetic changes associated with prostate cancer in humans. In *Cancer Surveys*, Vol 11, Isaacs, JT (ed.) pp 15-24. Cold Spring Harbor Laboratory Press: Cold Spring Harbor, NY.

ISRAELI RS, POWELL CT, FAIR WR AND HESTON WDW. (1993). Molecular cloning of a complementary DNA encoding a prostate-specific membrane antigen. *Cancer Res.*, **53**, 227-230.

ISRAELI RS, POWELL CT, CORR JG, FAIR WR AND HESTON WDW. (1994). Expression of the prostate-specific membrane antigen. *Cancer Res.*, **54**, 1807-1811.

KAIGN ME, NARAYAN KS, OHNUKI Y AND LECHNER JF. (1979). Establishment and characterization of a human prostatic carcinoma cell line (DU-145). *Invest. Urol.*, **17**, 16-23.

KAO FT, JONES C AND PUCK TT. (1976). Genetics of somatic mammalian cells: genetic, immunologic and biochemical analysis with Chinese hamster cell hybrids containing selected human chromosomes. *Proc. Natl Acad. Sci. USA*, **73**, 193-197.

KEER WP, KOSLOWSKI JM, TSAI YC, LEE C, MCEWAN RN AND GRAYHACK JT. (1990). Elevated transferrin receptor content in human prostate cancer cell lines assessed *in vitro* and *in vivo*. *J. Urol.*, **143**, 381-385.

KOCH J, FISCHER H, ASKHOLM H, HINDLEJAER H, PEDERSEN S, KALVRAA S AND BOLUND L. (1993). Identification of a supernumerary der (18) chromosome by a rational strategy for the cytogenetic typing of small marker chromosomes with chromosome-specific DNA probes. *Clin. Genet.*, **43**, 200-203.

LANDEGENT JE, JANSEN IN DE WAL N, DIRKS RW, BAGO F AND VAN DER PLOEG M. (1987). Use of whole cosmid cloned genomic sequences for chromosomal localisation by non-radioactive in situ hybridisation. *Hum. Genet.*, **77**, 366-370.

LONG AA, KOMMINOTH P, LEE E AND WOLFE HJ. (1993). Comparison of indirect and direct in-situ polymerase chain reaction in cell preparations and tissue sections. Detection of viral DNA, gene rearrangements and chromosomal translocations. *Histochemistry*, **99**, 151-162.

LOPES AD, DAVIS WL, ROSENTRAUS MJ, UVEGES AJ AND GILMAN SC. (1990). Immunohistochemical and pharmacokinetic characterization of the site-specific immunoconjugate CYT-356 derived from antiprostate antibody 7E11-C5. *Cancer Res.*, **50**, 6423-6429.

PARKS GD AND LAMB RA. (1991). Topology of eukaryotic type II membrane proteins: importance of *N*-terminal positively charged residues flanking the hydrophobic domain. *Cell*, **64**, 777-787.

PINKEL D, STRAWME T AND GRAY JW. (1986). Cytogenetic analysis using quantitative, high-sensitivity, fluorescence hybridisation. *Proc. Natl Acad. Sci. USA*, **83**, 2934-2939.

ROCHON YP, HOROSZEWICZ JS, BOYNTON AL, HOLMES EH, BARREN RJ, ERICKSON SJ, KENNY GM AND MURPHY GP. (1994). Western blot assay for prostate-specific membrane antigen in serum of prostate cancer patients. *Prostate*, **25**, 219-223.

ROSSI MC AND ZETTER BR. (1992). Selective stimulation of prostatic carcinoma cell proliferation by transferrin. *Proc. Natl Acad. Sci. USA*, **89**, 6197-6201.

STONE KR, MICKEY DD, WUNDERLI H, MICKEY GH AND PAULSON DF. (1978). Isolation of a human prostate carcinoma cell line (DU-145). *Int. J. Cancer*, **21**, 274-281.

TROYER JK, QI F, BECKETT ML, MORNINGSTAR MM AND WRIGHT GL. (1993). Molecular characterization of the 7E11-C5 prostate tumor-associated antigen. *J. Urol.*, **149**, 333A.

TROYER JK, ADAM M AND WRIGHT GL. (1994). Subcellular localisation of the 7E11-C5 prostate specific antigen. *Proc. Am. Assoc. Cancer Res.*, **35**, 283.

WYNANT GE, MURPHY GP, HOROSZEWICZ JS, NEAL CE, COLLIER BD, MITCHELL E, PURNELL G, TYSON I, HEAL A, ABDEL-NABI H AND WINZELBERG G. (1991). Immunoscintography of prostatic cancer: preliminary results with $^{111}$In-labelled monoclonal antibody 7E11-C5.3 (CYT-356). *Prostate*, **18**, 229-241.

YAN G, FUKABORI Y, MCBRIDE G, NIKOLAROPOLOUS S, AND MCKEEHAN WL. (1993). Exon switching and activation of stromal and embryonic fibroblast growth factor (FGF)-FGF receptor genes in prostate epithelial cells accompany stromal independence and malignancy. *Mol. Cell. Biol.*, **13**, 4513-4522.